# Side effect concerns and their impact on women’s uptake of modern family planning methods in rural Ghana: a mixed methods study

**DOI:** 10.1186/s12905-020-0885-0

**Published:** 2020-03-20

**Authors:** Leah A. Schrumpf, Maya J. Stephens, Nathaniel E. Nsarko, Eric Akosah, Joy Noel Baumgartner, Seth Ohemeng-Dapaah, Melissa H. Watt

**Affiliations:** 1 Duke Global Health Institute, Duke University, Box 90519, Durham, NC 27708 USA; 2 Millennium Promise Ghana, 14 Bathur St, East Legon, Accra Ghana

**Keywords:** Ghana, Family planning, Contraceptives, Barriers, Side effects

## Abstract

**Background:**

Despite availability of modern contraceptive methods and documented unmet need for family planning in Ghana, many women still report forgoing modern contraceptive use due to anticipated side effects. The goal of this study was to examine the use of modern family planning, in particular hormonal methods, in one district in rural Ghana, and to understand the role that side effects play in women’s decisions to start or continue use.

**Methods:**

This exploratory mixed-methods study included 281 surveys and 33 in-depth interviews of women 18–49 years old in the Amansie West District of Ghana between May and July 2018. The survey assessed contraceptive use and potential predictors of use. In-depth interviews examined the context around uptake and continuation of contraceptive use, with a particular focus on the role of perceived and experienced side effects.

**Results:**

The prevalence of unmet need for modern family planning among sexually active women who wanted to avoid pregnancy (*n* = 135) was 68.9%. No factors were found to be significantly different in comparing those with a met need and unmet for modern family planning. Qualitative interviews revealed significant concerns about side effects stemming from previous method experiences and/or rumors regarding short-term impacts and perceived long-term consequences of family planning use. Side effects mentioned include menstrual changes (heavier bleeding, amenorrhea or oligomenorrhea), infertility and childbirth complications.

**Conclusion:**

As programs have improved women’s ability to access modern family planning, it is paramount to address patient-level barriers to uptake, in particular information about side effects and misconceptions about long-term use. Unintended pregnancies can be reduced through comprehensive counseling about contraceptive options including accurate information about side effects, and the development of new contraceptive technologies that meet women’s needs in low-income countries.

## Background

Modern family planning methods are a cost-effective strategy for reducing high-risk pregnancies, decreasing unsafe abortions, and allowing for birth spacing and limiting [1–4]. Despite advances in contraceptive technology and availability, 214 million women had an unmet need for modern family planning in 2017 [5].

In order to inform the delivery of family planning services, it is important to understand the factors and characteristics that contribute to a woman’s decision to use modern family planning. Demographic factors influencing family planning use may include age, family size, distance from a health care facility and education level [6, 7]. Additionally, family planning use is influenced by women’s norms and perceptions. Women may face cultural or religious pressures against using family planning, often rooted in beliefs that family planning leads to unfaithfulness or interferes with goals of procreation [7, 8].

Side effects of modern family planning methods, either experienced or anticipated, have been identified as a common reason that women either choose not to start or discontinue contraceptives. Side effects include menstrual changes (heavier bleeding, amenorrhea or oligomenorrhea), changes in weight, headaches, dizziness, nausea, and cardiovascular impacts. In addition, women may harbor fears of long-term effects of contraceptive use, such as infertility and childbirth complications [8, 9]. A 2014 systematic review found a significant proportion of women attributed their unmet need for family planning to a fear of side effects: 28% in Africa, 23% in Asia, and 35% in Latin America and the Caribbean [10]. A fear of side effects may occur when a woman or someone she knows has experienced side effects with a method, or when rumors or overestimations or rare complications are considered factual [7, 8, 11–13].

Ghana has historically had one of the highest rates of unmet need for family planning in Africa, despite having a relatively strong family planning program. Ghana’s rate of unmet need among married women is 32.9 whereas many surrounding countries have a lower rate of unmet need among married women including Senegal (26.2), Nigeria (23.7) and Cote d’Ivoire (30.9) [14]. Family planning methods are available at both private and public healthcare facilities and offer a diverse contraceptive mix, including injectables, implants and hormonal birth control pills [8]. The Amansie West district has 22 public health facilities, comprised of 6 health centers and 16 Community-based Health Planning and Services compounds, 5 private health facilities and 1 hospital. Within these various health facilities modern family planning methods (pills, intrauterine devices (IUDs), and implants) can be administered by trained medical doctors, midwives, and trained Community Health Officers. Nurses in health facilities are able to administer pills and condoms. Outside of health facilities condoms, pills, and injectables are available at pharmacies and drug shops [15, 16].

Despite efforts to make contraceptives accessible, about one-third of married women have an unmet need for family planning [17]. Although the use of modern family planning methods has increased from 5 to 22% between 1988 and 2014, one in four contraceptive users discontinued use within the first year. The main reason reported for discontinuing injectables and implants were side effects or other health concerns and health concerns as a reason for non-use of modern methods in Ghana has been growing over time [12, 17].

This study was conducted with the goal of understanding modern family planning use in a rural setting of Ghana with three aims. First, we aimed to estimate the prevalence of modern family planning use and the prevalence of unmet need for modern family planning. Second, we identified factors associated with unmet need for modern family planning use, including factors at an individual, household and health care level. Lastly, we sought to qualitatively examine and understand women’s experiences with choices and behaviors related to family planning use, with a focus on the role of side effects. This data can help inform the delivery of modern contraceptives to all women wanting to delay or limit their pregnancies.

## Methods

### Setting

This exploratory mixed-methods study was conducted in the Amansie West District, in the Ashanti Region of Ghana. The population of the area is almost entirely rural (95.6%), with an estimated population of 149,437 in 2014 and annual growth of 2.7% [18, 19].

### Sampling

The study included 281 household surveys and 33 in-depth interviews of women 18–49 years old from six subdistricts of the Amansie West District. Data were gathered from May to July 2018 as part of a larger study examining the role of community health workers (CHWs) in family planning use. Six of the seven subdistricts within the Amansie West District were selected based on accessibility and penetration of the national CHW program. The six sub-districts were divided into 11 geographical zones containing at least 100 women of reproductive age (18–49) whose household was registered by a CHW. An average of 30 women were recruited from each zone in order to have geographic representation in our sample; the final sample size was informed by the resources available to us in this study. A subset of individuals who completed the household survey were purposively sampled to participate in a separate in-depth interview. Participants for the in-depth interview were selected based on current, past or lack of modern method use.

### Procedures

To collect the survey data, a team of six female research assistants, bilingual in English and Twi, were trained in ethics and research procedures. The research assistants approached women in their homes to tell them about the study and invite them to participate. After written informed consent, the research assistant administered the structured interview using an electronic tablet, which took approximately 45 min. At the end of the interview, participants were asked if they might be interested in taking part in a subsequent in-depth interview; if yes, then their contact information was collected to schedule the interview at a later time.

To conduct in-depth interviews (IDIs), three bilingual nurses from the district were trained on research ethics and qualitative research. IDIs were scheduled in participants’ homes at a time that was convenient and maximized privacy. Participants were provided a separate written informed consent for the IDI, which included consent for audio recording. IDIs were conducted in Twi and lasted on average 30 min. Following each interview, field notes were written and then later reviewed with the full research team.

### Instruments

#### Structured survey

The structured survey was created based on a review of the literature and consultation with local public health professionals. The survey was locally translated into Twi and reviewed by multiple individuals to confirm accurate translation. The survey was pre-tested in a rural community prior to data collection, which resulted in slight modifications.

The survey included the following constructs: demographics; pregnancy history; knowledge and perceived availability of various forms of contraceptives; use of contraceptives; pregnancy intention and attitudes towards pregnancy (α = 0.81); depression PHQ-9 (α = 0.74); autonomy (α = 0.77); partner communication (α = 0.74); freedom from coercion (α = 0.80); and partner support (α = 0.80) [20–24].

#### In-depth interviews

The in-depth interviews were conducted using a semi-structured guide that included open-ended questions and probes to explore community and individual perspectives of family planning, barriers to use, experiences with family planning use, and reasons for using or not using family planning. The interview guide developed for this study, available as a supplementary file, was reviewed by local public health professionals and pretested in the community. Research assistants translated the guide into Twi during the interviews to adjust the phrasing for a natural, casual conversation.

### Data analysis

Survey data were analyzed using R Studio. In order to define a population that could be in need of community-based contraception, we excluded individuals who were not sexually active (defined as three months since last sex), currently pregnant, wished to become pregnant in the next few months, or reported being infertile (includes hysterectomy) from the analysis. This resulted in a sample for analysis of 135 women. While definitions of unmet need for population level analyses typically include women, who have unwanted/mistimed pregnancies the parent study was particularly interested in community level modern family planning method gaps that might be facilitated by community health workers. We were also most interested in highly effective modern methods and thus current use of natural and barrier methods were also excluded from our main analyses. Individuals were classified as having an unmet need for modern contraception if they met the criteria for inclusion but reported that they were not using a hormonal method (pills, injectables, implants), female sterilization, male sterilization or an IUD. After examining descriptive statistics, bivariate analysis explored whether key factors were significantly associated with unmet need for these highly effective modern family planning methods. Because bivariate statistics were not significant, multivariate statistics were not used.

Qualitative analysis was conducted using applied thematic analysis [25]. Audio recordings were simultaneously translated and transcribed in English. NVivo 12 was used to facilitate the organization and coding of transcripts. Emergent themes were identified through an iterative process of summary memos and open coding, which led to the development of a structured codebook. Overarching domains were created as parent codes, and child codes were used to organize emerging themes. Coded texts were reviewed and synthesized, and representative quotes were identified to capture meaning and provide context.

## Results

### Demographics

Table 1 summarizes the demographics of the sub-sample of participants who had a current need for modern family planning (*n* = 135). On average, participants were 29.4 years of age. About half (45%, *n* = 61) were married. In this community couples who have undergone customary marital rights or those that are living together with children were considered to be married. Half of the participants (52%, *n* = 70) had three or more children. Education was low, with only 15.6% of participants reporting any secondary school education.

**Table 1:** Sample demographics and characteristics (*n* = 135)

	Mean	SD
Age	29.4	7.8
	n	%
Education level		
No Education	15	11.1%
Primary	42	31.9%
Middle	57	43.0%
Secondary and above	21	15.6%
Marital Status		
Married	61	45.2%
Living with partner	55	40.7%
In a relationship but not living together	19	14.1%
Single	2	1.5%
Religion		
Christianity	110	81.5%
Islam	5	3.7%
Not religious	2	1.5%
Other	18	13.3%
Number of Children		
0	9	6.7%
1	22	16.3%
2	34	25.2%
3+	70	51.9%

### Family planning use

Considering the family planning needs of the sample, 31.1% (*n* = 42) had a met need, and 68.9% (*n* = 93) had an unmet need. More than half (*n* = 23) of women with a met need were using the injectable, Depo-Medroxyprogesterone (DMPA) (Table 2). In the bivariate analysis of factors potentially associated with family planning use (i.e., pregnancy intentions and attitudes, depression, level of autonomy, communication with their partner, freedom from coercion and levels of partner support), none of the measures were significantly associated (Table 3).

**Table 2:** Modern contraceptive use (*n* = 135)

Method	n	%
No use of modern methods	93	68.9
Use of modern methods	42	31.1
Injectables		
DMPA (3 months)	23	
EV/NETE (1 month)	2	
Implants		
Levonorgestrel (5 yrs)	8	
Etonogestrel (3 yr)	2	
IUD	0	
Pills	7	
Female Sterilization	1	
Male Sterilization	0

**Table 3:** Predictors of unmet need for modern family planning (*n* = 135)

	Overall	Unmet need n (%)	Unadjusted OR (95% Cl)	*p*-value
Depression				
No Depressive Symptoms	60	40 (66.7%)	REF	–
Depressive Symptoms	75	53 (70.1%)	1.2 (0.58–2.50)	0.62
Attitudes Towards Pregnancy				
Positive Attitude	95	65 (68.4%)	REF	–
Negative Attitude	39	27 (69.2%)	1.04 (0.46–2.32)	0.93
Autonomy				
Autonomy	133	–	0.99 (0.86–1.13)	0.84
Partner Components				
Communication	130	–	1.02 (0.87–1.20)	0.78
Freedom from Coercion	133	–	1.04 (0.91–1.19)	0.54
Partner Support	134	–	1.01 (0.90–1.14)	0.83

### Qualitative insights on family planning use

In the qualitative data, two prominent themes emerged to explain unmet need: concerns about side effects and misconceptions about the long-term effects of family planning (Table 4).

**Table 4:** Barriers to uptake and continuation of modern family planning methods

Concerns about side effects	
Loss of menstruation	*“What I have heard people say is that when you do family planning, you will not menstruate. God created women to menstruate every month, but because of the use of family planning people are not menstruating and that is what is causing the dizziness and tiredness when you walk a short distance. That is what some people are saying.” (Woman with Met need, 20–29 years old)*
	*“Some also say you can die early because you are not able to menstruate.” (Woman with Unmet Need, 20–29 years old)*
Excessive menstruation	*“I stopped using it because I was menstruating every two weeks. I thought I was developing some sickness, so I became afraid and stopped using it. Every two weeks when I was menstruating, I bled for so long without it stopping, sometimes throughout the two weeks, so I became afraid and stopped using it.” (Woman with Unmet Need, 30–39 years old)*
Sickness and physical impacts	*“When I go to my friend, and she suggests we go and do family planning, I tell her no because they say when you do it, you become sick and dizzy. Some also say that when you do the family planning you lose so much weight and you become shabby looking … (My partner) always tells me to do the family planning, but I just didn’t want to do it. (Woman with Unmet Need, 20–29 years old)*
	I haven’t regretted using family planning, but I was constantly getting sick after doing the 3-month injectable method. So, I stopped and got pregnant with my lastborn because I knew it was the 3-month injectable that was making me sick. But after that birth, I began using the 1-month injectable method.” (Woman with Met Need, 40–49 years old)
Misconceptions of long-term impacts	
Death	*“Some also say you can die early because you are not able to menstruate. When the blood cannot come out, it settles in your abdomen and can kill you. And some also say you can become barren or that family planning renders you incapable of having more children.” (Woman with Unmet Need, 20–29 years old)*
	*“There are also rumors in this community that someone went to do the implant and it is said the implant got lost in her bloodstream and she died.” (Woman with Unmet Need, 30–39 years old)*
Infertility	*“When I decided to do the family planning, some people told me that you will be able to space your births and your children, but when you decide to give birth, it will be impossible.” (Woman with Met Need, 20–29 years old)*

#### Side effects

Side effects were mentioned as a potential concern in all qualitative interviews. For many, the concerns about side effects outweighed the perceived benefits of using family planning. Of the 17 participants who had discontinued family planning use, only 5 reported experiencing side effects themselves, while the majority recited side effects they believed were associated with modern family planning use. The most common concern about hormonal contraceptives was the resultant changes in menstrual patterns. There was a belief that menstruation was a means of cleansing the body, and concerns that a lack of menstruation could lead to sickness, dizziness, bloating, and fainting. Additionally, amenorrhea was concerning for women because they could no longer monitor whether or not they were pregnant.

In addition to changes in menstruation, participants mentioned other side effects they were concerned about, including sickness, dizziness, and changes in weight. Reduction in weight was seen as an undesirable side effect, while weight gain was seen as a desirable side effect. The seven participants currently using DMPA had experienced at least one of these side effects. Even in cases where participants reported support from their partner, family or religious community to use family planning, anxiety about side effects deterred them from using family planning—support was not enough to overcome what the women articulated as unacceptable side effects. Women who had not experienced side effects themselves discussed side effects as the most common reason that other women did not use modern family planning.

#### Misconceptions about long-term impacts

Participants both using and not using a modern contraceptive method reported misconceptions in the community, particularly about hormonal methods. The most common misconceptions were rumors about the long-term adverse effects caused by modern family planning. Women recited rumors that family planning use led to fibroids, infertility, birth complications, and even premature death. In most cases, these long-term impacts were attributed to changes in menstrual patterns, typically associated with injectables and implants.

Three participants discussed rumors that implants caused fainting and death due to a restriction in blood flow. The rumors and misconceptions that were reported about family planning use spanned all 14 communities that were included in the qualitative portion of the study, illustrating the ubiquitous nature of these concerns.

## Discussion

Knowledge of modern family planning methods is high throughout Ghana; nationwide, 99% of women with an unmet need for family planning identified at least one modern method [17]. Despite high levels of knowledge, we found that among 135 women who were sexually active and wanting to avoid pregnancy, a majority (68.9%) had an unmet need for a highly effective modern family planning method. When examining factors that might explain unmet need, no significant associations were identified. Our qualitative data suggests that fear of side effects and misconceptions about family planning methods is likely driving the gap between knowledge and behavior in family planning use. This study suggests a need to address accurate information about family planning methods, especially injectables the most common form of modern family planning in African countries [26]. Addressing structural barriers of access to contraceptives will be insufficient if misinformation about side effects and long-term adverse effects persist.

In order to meet the needs of women who wish to postpone or limit their pregnancies, it is important to have targeted interventions to address the fears and concerns caused by menstrual bleeding changes that frequently occur with hormonal methods such as injectables and implants. This is especially important in low- and middle-income countries (LMICs) where the use of injectables is being prompted due to its higher effectiveness level, as compared to oral hormonal pills, and the ability to use the product discretely [27, 28]. The universal concern about menstruation in our sample demonstrates the need for improved counseling, before and during use, to educate women about the role of menstruation in reproduction and how hormones impact menstrual patterns [8, 12, 13]. Both uptake and continuation of reversible contraceptives requires regular counseling, scheduled follow-up, and clinical management of contraceptive side effects [29]. Health workers, including CHWs, involved in providing family planning education and provision should be equipped to provide reproductive education to help women understand and differentiate between nonharmful and harmful side effects. FHI360 has developed a job aid called “NORMAL” to help health workers counsel clients on expected changes of menstruation on various forms of hormonal contraception [9]. There is evidence that job aids with accurate injectable information have been shown to increase injectable use in low-resource settings and could be adapted to the Ghanaian context [30]. CHWs need additional training to help women understand and manage side effects from modern family planning. Job aids such as “NORMAL” could provide CHWs with a tool to better counsel and manage clients regarding uptake and continuation of family planning methods. Comprehensive counseling, including accurate information on side effects, has been shown to increase continuation of modern methods [31, 32]. Additional qualitative research among different communities is needed to understand the beliefs that underlie women’s concerns about menstrual changes. Better understanding these beliefs can inform culturally congruent counseling approaches to promote the uptake and sustainability of family planning methods.

Certain side effects will always be considered unacceptable for some women, making their family planning options more limited. A long-term solution for family planning coverage requires investments in new contraceptive technologies that are responsive to women’s preferences and needs. This includes the development of both hormonal and nonhormonal long-acting reversible contraceptives that are accessible and effective in low-income settings. Several new contraceptive technologies under development may hold promise, including biodegradable implants, longer-acting injectables, IUDs that are easier to insert, and non-hormonal vaginal rings [33, 34]. Hopefully, these new technologies will address some barriers to family planning use and provide more options for women and couples to limit or space their pregnancies.

The study findings must be interpreted in the context of the study’s limitations. First, social desirability biases may be present in the survey results. The in-depth interviews were conducted by local nurses responsible for administering family planning methods in the clinics; therefore, participants may have been less likely to speak negatively about services or areas in which the nurses work. It is important to note variation among these interviews, as some participants were more willing to discuss and share than others. Second, the in-depth interviews were simultaneously translated and transcribed from the local language, Twi, into English; therefore, some details and phrasing may have been lost in the process. Third, the sample size for the survey was not powered to detect statistically significant differences. Lastly, we did not explore more deeply whether women felt their family planning needs were being met via barrier and/or natural methods—although less effective, some women and couples purposefully choose this option.

## Conclusion

Even as modern contraceptives become increasingly accessible, women may perceive potential drawbacks of highly effective family planning methods to outweigh the benefits. The future of family planning research and implementation should focus on developing and implementing evidence-based counseling tools to promote the uptake and continuation of the current method mix and investing in the development of new family planning technologies that fit the lifestyles and needs of women in LMICs.

## Data Availability

The data and all related study materials may be requested from the faculty mentor on this study (Dr. Melissa Watt, melissa.watt@duke.edu).

## Abbreviations

CHW: Community Health Worker
DHS: Demographic Health Survey
DMPA: Depo-Medroxyprogesterone
EV/NETE: Estradiol valerate/norethisterone enantate
IDI: In-depth interview
IUD: Intrauterine device
LMICs: Low- and middle-income countries
